# Silicon Supplementation Alleviates the Salinity Stress in Wheat Plants by Enhancing the Plant Water Status, Photosynthetic Pigments, Proline Content and Antioxidant Enzyme Activities

**DOI:** 10.3390/plants11192525

**Published:** 2022-09-26

**Authors:** Pooja Singh, Vikram Kumar, Jyoti Sharma, Sakshi Saini, Priyanka Sharma, Sandeep Kumar, Yogesh Sinhmar, Dhirendra Kumar, Asha Sharma

**Affiliations:** 1 Department of Botany, Maharshi Dayanand University, Rohtak 124001, Haryana, India; 2 Department of Botany, Baba Mastnath University, Rohtak 124001, Haryana, India; 3 Department of Botany, Kurukshetra University, Kurukshetra 136119, Haryana, India; 4 Department of Botany, Chaudhary Bansi Lal University, Bhiwani 127021, Haryana, India

**Keywords:** wheat, silicon, salt stress, KRL-210, WH-1105, phenol content, electrolyte leakage

## Abstract

Silicon (Si) is the most abundant element on earth after oxygen and is very important for plant growth under stress conditions. In the present study, we inspected the role of Si in the mitigation of the negative effect of salt stress at three concentrations (40 mM, 80 mM, and 120 mM NaCl) in two wheat varieties (KRL-210 and WH-1105) with or without Si (0 mM and 2 mM) treatment. Our results showed that photosynthetic pigments, chlorophyll stability index, relative water content, protein content, and carbohydrate content were reduced at all three salt stress concentrations in both wheat varieties. Moreover, lipid peroxidation, proline content, phenol content, and electrolyte leakage significantly increased under salinity stress. The antioxidant enzyme activities, like catalase and peroxidase, were significantly enhanced under salinity in both leaves and roots; however, SOD activity was drastically decreased under salt stress in both leaves and roots. These negative effects of salinity were more pronounced in WH-1105, as KRL-210 is a salt-tolerant wheat variety. On the other hand, supplementation of Si improved the photosynthetic pigments, relative water, protein, and carbohydrate contents in both varieties. In addition, proline content, MDA content, and electrolyte leakage were shown to decline following Si application under salt stress. It was found that applying Si enhanced the antioxidant enzyme activities under stress conditions. Si showed better results in WH-1105 than in KRL-210. Furthermore, Si was found to be more effective at a salt concentration of 120 mM compared to low salt concentrations (40 mM, 80 mM), indicating that it significantly improved plant growth under stressed conditions. Our experimental findings will open a new area of research in Si application for the identification and implication of novel genes involved in enhancing salinity tolerance.

## 1. Introduction

Wheat (*Triticum aestivum*) is among the major staple crops produced worldwide, mainly for human consumption [1]. Due to the increasing population, the world is highly dependent on wheat for food. Globally, wheat provides approximately 55% of the carbohydrates and 20% of the calories consumed on a daily basis [2]. Various environmental factors affect crop production, with salinity being one of them. Salinity is a crucial form of abiotic stress that badly affects food crops [3,4]. Due to human activities and climate change, salinity affects around one fifth of cultivated areas and a third of all irrigated agricultural land area on which staple crops such as wheat are grown; this rate is increasing steadily [5]. However, research is ongoing to improve crop production under these stresses. Salt stress causes physiological, biochemical, and metabolic alterations in plants, resulting in poor crop production. Under high-stress conditions, the water uptake is restricted in the plants from the soil, affecting the water status of the whole plant [6,7]. A reduction in water uptake results in decreased stomatal conductance and increased transpiration and hinders cell growth [8]. In addition, salt stress negatively affects crop production by damaging the photosynthetic apparatus and inhibiting the ribulose-1,5-bisphosphate enzyme, which helps in photosynthetic pigment formation like chlorophyll and carotenoids [9].

Consequently, salt stress degrades the photosynthetic pigments in plants, resulting in chlorosis, necrosis, and early senescence, as well as many other important changes that ultimately slow plant growth [10]. Salt stress for a long period causes severe ionic and oxidative stresses, due to the over-absorption of NaCl in plants [11]. These stresses damage plant growth [10]. Salt stress causes an elevation in the reactive oxygen species (ROS) content, e.g., hydrogen peroxide (H_2_O_2_) and superoxide (O_2_^−^), in plant cells, which hinders cellular expansion [12]. Oxidative stress is a vital indicator of salinity stress, which results from an imbalance between ROS and antioxidants (e.g., superoxide dismutase (SOD), peroxidase (POX), catalase (CAT)) contents. Under optimal conditions, plant cells maintain a balance between ROS and antioxidants, i.e., a redox balance [13,14]. The formation of an excess of ROS restricts the absorption of nutrients in plants and destroys important macromolecules like nucleic acids, proteins, and membrane lipids, which leads to degraded membrane integrity [15]. As a result, the significant production of ROS exceeds the scavenging rate [9]. Plants accumulate high amounts of compatible solute compounds or osmoprotectants such as proline, glycine betaine, amino acids, sugar, and phenolic compounds in order to cope with the salinity stress conditions [16,17].

On the other hand, plants have developed defense mechanisms to diminish the negative consequences of salinity, i.e., by accumulating various antioxidant enzymes like POX, SOD, APX, and CAT, along with non-enzymatic antioxidants like tocopherol and carotene, and glutathione [18]. Under various stress conditions, plants produce a high content of ROS, which generate oxidative stress, while antioxidant enzymes provide protection against these oxidative stress conditions [8,19]. This self-defense system in plants is not enough to overcome the negative impact of salinity; thus, there is a great need to counter this negative effect through different approaches, and Si application is one of them.

Silicon is one of the most abundant elements in the earth’s crust, comprising 28% of the lithosphere. The International Plant Nutrition Institute (IPNI) has termed Si as a “quasi-essential” element, recognizing its importance in plants under stress conditions [20]. The application of Si helps maintain a plant’s water status by depositing a silica film on the epidermis of the leaf, thereby reducing transpiration and increasing the photosynthetic rate [8]. According to Almeida et al. [21], supplementation of Si has been recognized as an environmentally eco-friendly technique that enhances antioxidant enzyme activities and maintains ROS formation, ionic balance, and K⁺/Na⁺ ratio in plants under salinity. Si is established as a multivalent element that not only reduces salinity stress but also reduces various abiotic and biotic stresses in plants and improves crop production by depositing in the plant cells as amorphous silica [22]. It has been stated that Si supplementation enhances the antioxidant defense mechanism, relative water content (RWC), and nutrient balance and decreases the electrolyte leakage in wheat plants under saline conditions [23]. Si was also found to be effective at improving the morphological characteristics, e.g., biomass and plant length, of sunflower and sorghum plants under high salinity conditions [24,25]. In chickpea plants, Si as a fertilizer reduces the translocation of Na^+^ from root to shoot and leaves under salt stress [26]. Si mediated stress tolerance in wheat, increased absorption of K^+^, decreased the uptake of Na^+^ and enhanced the chlorophyll ratio, plant weight, and antioxidant enzyme activities, resulting in better plant growth under salinity [27]. 

Wheat is a Si-accumulator that uptakes Si through the roots when it is present in an available form in soil [28]. Thus, considerable research has been conducted to determine the role of Si supplementation on the regulation of antioxidant enzymes, proline content, lipid peroxidation, phenol content, carbohydrate content, electrolyte leakage and RWC, as well as the photosynthetic pigments of two wheat genotypes under different NaCl stress conditions.

## 2. Results

### 2.1. Effect of Si on RWC and Electrolyte Leakage of Leaves and Roots under Salinity

**Figure 1:**
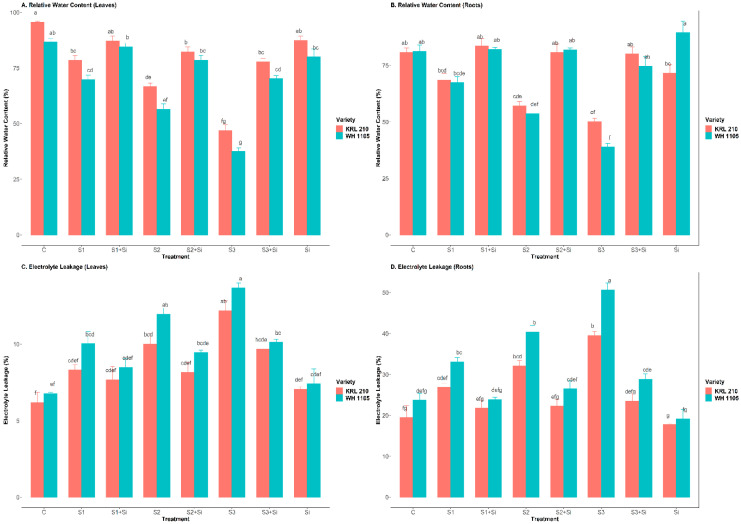
Effect of Si on relative water content (RWC) of leaves (**A**), RWC of roots (**B**), electrolyte leakage of leaves (**C**), and electrolyte leakage of roots (**D**) in salt-stressed wheat plants. Means ± SE; Two-way ANOVA with Tukey’s post hoc test; Each bar having different alphabets was significantly different between treatments (*p* < 0.05). C: Control, S1: 40 mM NaCl stress, S2: 80 mM NaCl stress, S3: 120 mM NaCl stress, Si: 2 mM Silicon.

The RWC of leaves and roots was drastically reduced under increased salt stress (Figure 1A,B). Salinity affected both wheat varieties when the salt concentration increased from 40 mM NaCl to 120 mM NaCl. Compared to the control, the RWC of leaves was reduced by up to 51% in KRL-210 and 57% in WH-1105 at a high salt stress concentration (S3). Similarly, the RWC of root was reduced by up to 38% in KRL-210 and 52% in WH-1105 at high salinity (S3). Additionally, Si improved the RWC of leaves and roots by up to 66% and 60% (S3 + Si) in KRL-210 and 87.3% and 92% (S3 + Si) in WH-1105 against high saline stress (S3), respectively. Moreover, the electrolyte leakage of leaves and root was increased by up to 97% and 102% (S3) in KRL-210 and 102% and 113% (S3) in WH-1105 under high salinity conditions when compared to the control. The roots of both wheat varieties were badly affected when electrolyte leakage increased during salt stress (Figure 1C,D). However, Si supplementation partially alleviated the negative effect of salinity by decreasing the electrolyte leakage of leaves and roots by up to 21% and 41% (S3 + Si) in KRL-210 and 26% and 43% (S3 + Si) in WH-1105 (S3). There was no significant effect of Si under non-stressed conditions in either variety.

### 2.2. Effect of Si on Chlorophyll ‘a’ and ‘b’ Content under Salinity

**Figure 2:**
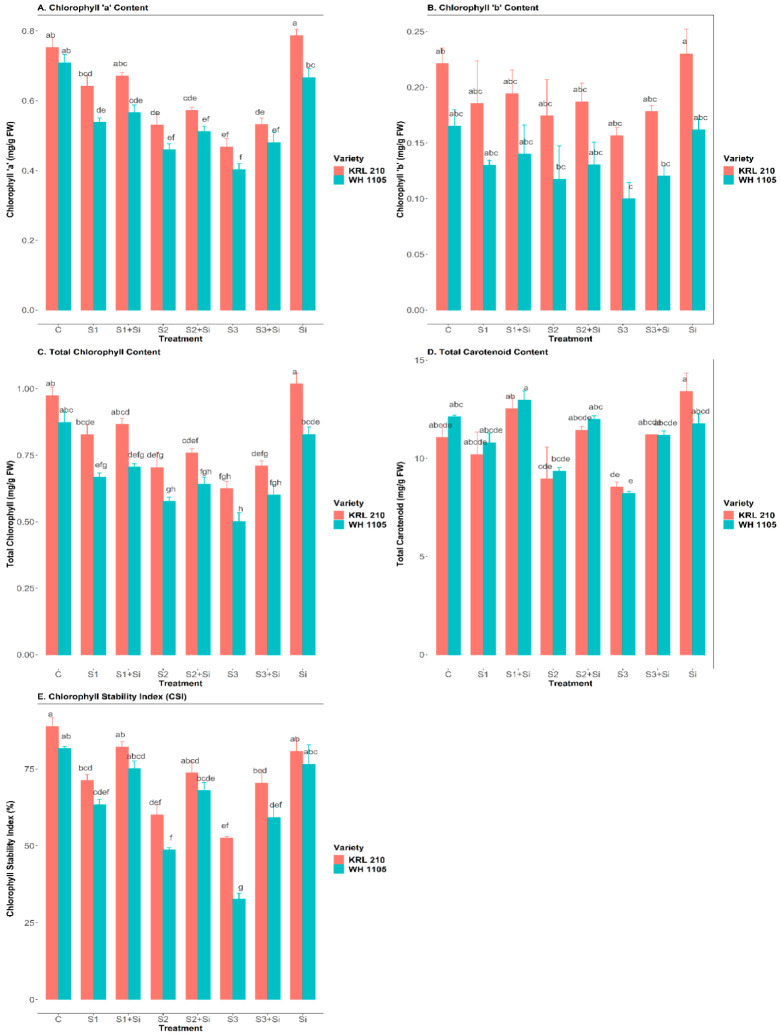
Effect of Si on chlorophyll ‘a’ (**A**), chlorophyll ‘b’ (**B**), total chlorophyll (**C**), carotenoid content (**D**), and CSI (**E**) in salt-stressed wheat plants. Means ± SE; Two-way ANOVA with Tukey’s post hoc test; Each bar having different alphabets was significantly different between treatments (*p* < 0.05). C: Control, S1: 40 mM NaCl stress, S2: 80 mM NaCl stress, S3: 120 mM NaCl stress, Si: 2 mM Silicon.

Our results showed that chlorophyll ‘a’ and chlorophyll ‘b’ were significantly reduced under a salinity treatment of 120 mM NaCl in both wheat varieties (Figure 2A,B). Chlorophyll ‘a’ declined from 0.642 ± 0.030 (S1) to 0.467 ± 0.024 mg/g FW (S3) in KRL-210 and 0.537 ± 0.012 (S1) to 0.402 ± 0.017 mg/g FW (S3) in WH-1105 variety under salinity compared to control plants (Figure 2A). Similarly, chlorophyll ‘b’ reduced from 0.185 ± 0.038 (S1) to 0.156 ± 0.006 mg/g FW (S3) in KRL-210 and 0.129 ± 0.004 (S1) to 0.99 ± 0.014 mg/g FW (S3) in WH-1105 compared to the control (Figure 2B). A more significant reduction was observed in WH-1105 than in KRL-210 under high-stress conditions (*p* < 0.05). However, supplementation of Si under saline stress improved the chlorophyll ‘a’ content by up to 0.532 ± 0.019 mg/g FW (S3 + Si) in KRL-210 and 0.480 ± 0.030 mg/g FW (S3 + Si) in WH-1105 (S3). Likewise, the addition of Si under salinity enhanced chlorophyll ‘b’ by up to 0.178 ± 0.005 mg/g FW (S3 + Si) in KRL-210 and 0.120 ± 0.009 mg/g FW (S3 + Si) in WH-1105 compared with salt stress condition alone (S3). Si overcame the decline rate in both varieties under stressed conditions. There was no significant effect of Si under non-stressed conditions in either variety.

### 2.3. Effect of Si on Total Chlorophyll, Carotenoid Content, and Chlorophyll Stability Index (CSI) under Salinity

The total chlorophyll and carotenoid contents were also reduced by up to 35% and 22% in KRL-210 and 42% and 31%, respectively, in WH-1105 variety at the highest NaCl stress concentration (S3) compared with the control (Figure 2C,D). Si application increased the total chlorophyll and carotenoid contents by up to 13.7% and 31% (S3 + Si) in KRL-210 and 19.5% and 35.7% (S3 + Si), respectively, in WH-1105 (S3) (*p* < 0.05). Likewise, CSI was also decreased from moderate stress (S1), i.e., from 19.7% to 41%, under high-stress conditions (S3) in KRL-210, and from and 22% (S1) to 60% (S3) in WH-1105 when compared with the control (Figure 2E). In addition, Si enhanced the CSI by up to 34% (S3 + Si) in KRL-210 and 81.6% (S3 + Si) in WH-1105 (S3). A significant increment was noted in CSI under high-stress conditions with the application of Si. There was no significant result of Si under non-stressed conditions in either variety.

### 2.4. Effect of Si on Total Proline, Total Phenol Content, and Lipid Peroxidation under Salinity

**Figure 3:**
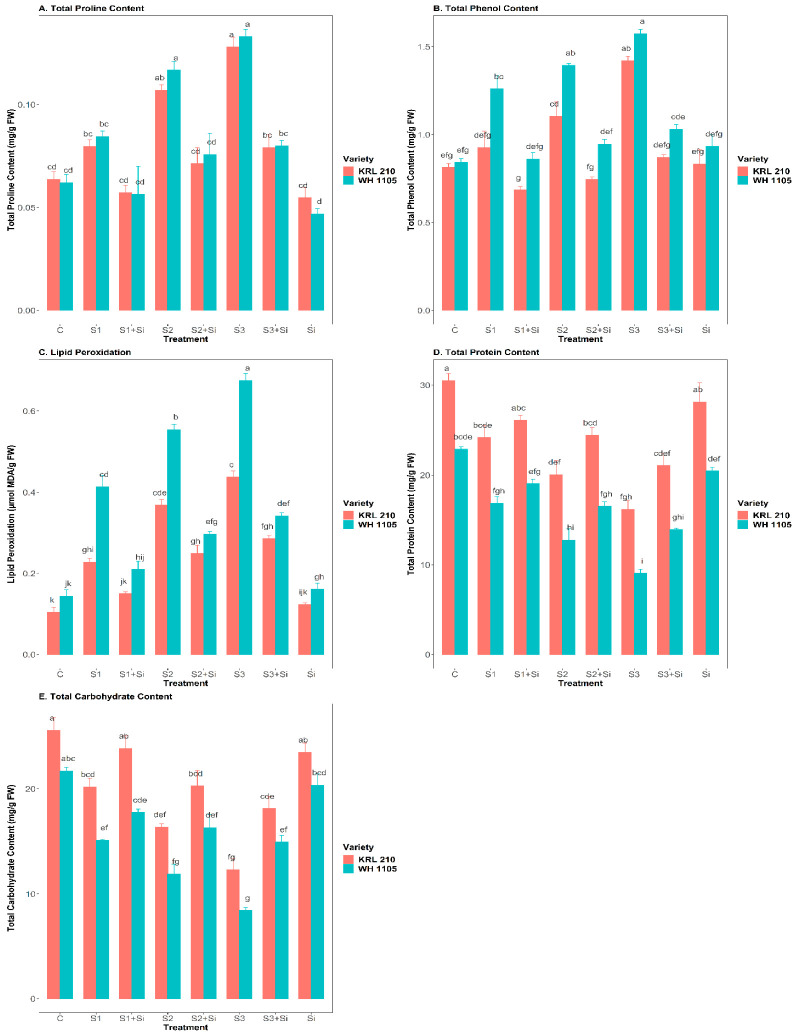
Effect of Si on total proline content (**A**), total phenol content (**B**), lipid peroxidation (**C**), total protein content (**D**), and total carbohydrate content (**E**) in salt-stressed wheat plants. Means ± SE; Two-way ANOVA with Tukey’s post hoc test; Each bar having different alphabets was significantly different between treatments (*p* < 0.05). C: Control, S1: 40 mM NaCl stress, S2: 80 mM NaCl stress, S3: 120 mM NaCl stress, Si: 2 mM Silicon.

In the present investigation, it was found that salinity increased the proline, phenol, and malondialdehyde (MDA) contents (Figure 3A–C) in both wheat varieties at all three levels of salinity (S1, S2, and S3). A substantial increase was observed in proline content with an increased salt concentration, i.e., up to 101% in KRL-210 and 114% in WH-1105 under high salinity stress (S3) compared to the control. The Si application decreased the rate of proline accumulation in plants by 38% in KRL-210 and 40% in WH-1105 under high saline conditions (S3 + Si). Likewise, the phenol content was also reduced by Si supplementation in stressed-treated wheat plants, which showed high phenol contents under salinity. Compared with the control, the phenol content was increased by up to 75% in KRL-210 and 86% in WH-1105 under high salinity stress (S3). However, Si reduced the phenol content to 39% in KRL-210 and 35% in WH-1105 under high salinity stress (S3 + Si). With increasing salt stress levels in plants, lipid peroxidation was also increased (S3). The average concentration of lipid peroxidation in control plants was 0.104 ± 0.012 µmol MDA g^−1^ FW in KRL-210 and 0.144 ± 0.015 µmol MDA g^−1^ FW in WH-1105, which increased to 0.437 ± 0.014 µmol MDA g^−1^ FW in KRL-210 and 0.675 ± 0.016 µmol MDA g^−1^ FW in WH-1105 with increased concentration of salinity (S3). However, Si supplementation reduced these values (S3) in both varieties to varying extents. WH-1105 was found to be more affected by the negative effects of salt stress than KRL-210.

### 2.5. Effect of Si on Total Protein and Total Carbohydrate Content under Salinity

Our results showed that salinity badly affected the total protein and carbohydrate contents when the NaCl concentration increased from 40 mM (S1) to 120 mM (S3) concentration (Figure 3D,E). In the control, the average concentration of protein was 30.52 ± 0.77 mg/g FW (C) in KRL-210 and 22.89 ± 0.25 mg/g FW (C) in WH-1105; these values reduced to 16.21 ± 0.99 mg/g FW in KRL-210 and 9.04 ± 0.46 mg/g FW in WH-1105, respectively, with increased salinity (S3). Meanwhile, with Si supplementation, the total protein content was increased by up to 21.06 ± 1.30 mg/g FW in KRL-210 and 13.94 ± 0.13 mg/g FW in WH-1105 under salt-stressed wheat plants (S3 + Si) when compared to salinity (S3). Similarly, a significant reduction was found in total carbohydrate content when the concentration of salinity increased. The maximum reduction was 52% in KRL-210 and 61% in WH-1105 under high saline conditions (S3) when compared with the control. Additionally, Si improved the carbohydrate content by up to 47% in KRL-210 and 77% in WH-1105 under stressed conditions (S3 + Si). However, there was no significant effect of Si under non-stressed conditions in either variety.

### 2.6. Effect of Si on the Antioxidant Enzymes Activity (CAT, POX, and SOD) under Salinity

**Figure 4:**
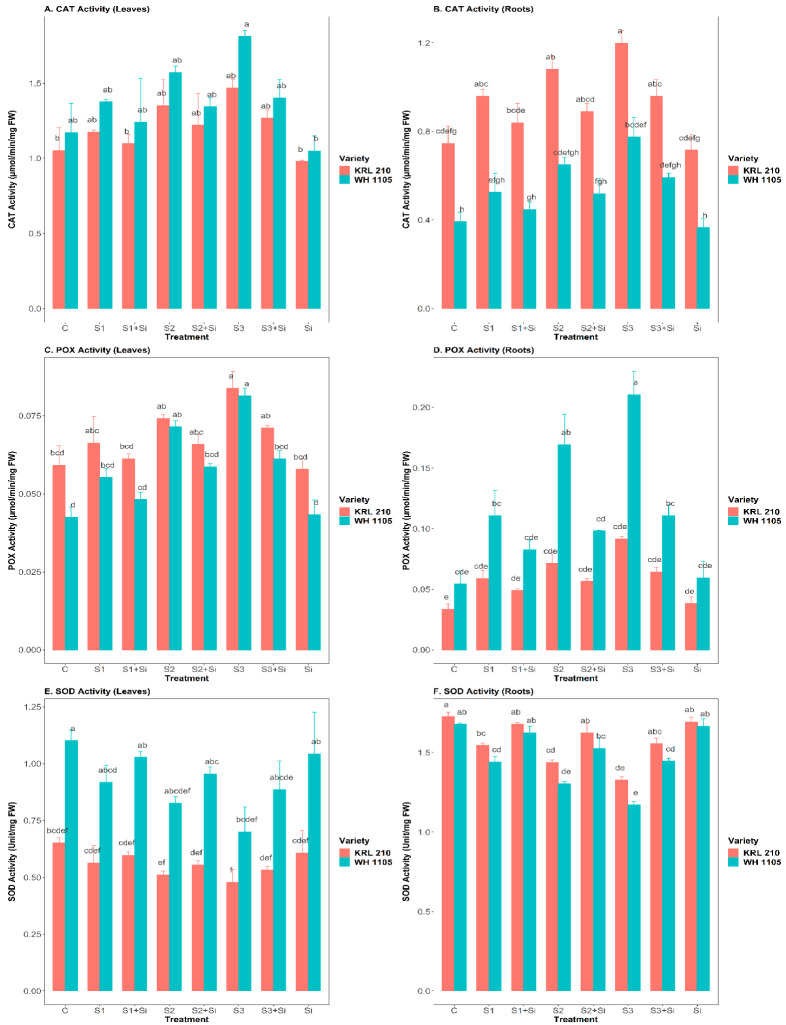
Effect of Si on catalase (CAT) activity of leaves (**A**), CAT activity of root (**B**), peroxidase (POX) activity of leaves (**C**), POX activity of root (**D**), superoxide dismutase (SOD) activity of leaves (**E**), and SOD activity of root (**F**) in salt-stressed wheat plants. Means ± SE; Two-way ANOVA with Tukey’s post hoc test; Each bar having different alphabets was significantly different between treatments (*p* < 0.05). C: Control, S1: 40 mM NaCl stress, S2: 80 mM NaCl stress, S3: 120 mM NaCl stress, Si: 2 mM Silicon.

Our evaluation revealed significant improvements in the enzyme activities of CAT and POX at all three-salinity levels (S1, S2, and S3). The CAT activity of leaves and roots was found to increase with increased salt stress level (Figure 4A,B), i.e., in leaves and roots, improvements of 39.4% and 60.5% in KRL-210 and 54.6% and 97% in WH-1105 were observed at the highest salt stress (S3) compared to the control. However, addition of Si reduced the CAT activity of leaves and roots by up to 14% and 20% in KRL-210 and 23% and 24% in WH-1105 under high salinity stress (S3 + Si). Similarly, the POX activity of leaves and roots also increased during stress conditions (Figure 4C,D) by up to 42% and 174% in KRL-210 and 91% and 288% in WH-1105 under high salt stress (S3) when compared with control. Si application reduced the rate of POX in leaves and roots by up to 15.2% and 30% in KRL-210 and 25% and 47.3% in WH-1105 under high salinity stress (S3 + Si). The roots of both wheat varieties showed higher activity (CAT and POX) than the leaves under stressed conditions. Supplementation of Si significantly improved the CAT and POX activity in leaves and roots of both wheat varieties. In contrast, SOD activity was observed to be greatly reduced in leaves and roots of stressed, treated wheat plants (Figure 4E,F). SOD activity decreased in leaves and roots by up to 26.5% and 23% in KRL-210 and 36.4% and 30.2% in WH-1105 under high salinity (S3) when compared with the control. Although Si treatment enhanced these values to a greater level under stress conditions, there was no significant effect of Si under non-stressed conditions in either variety.

### 2.7. Correlation Analysis

**Figure 5:**
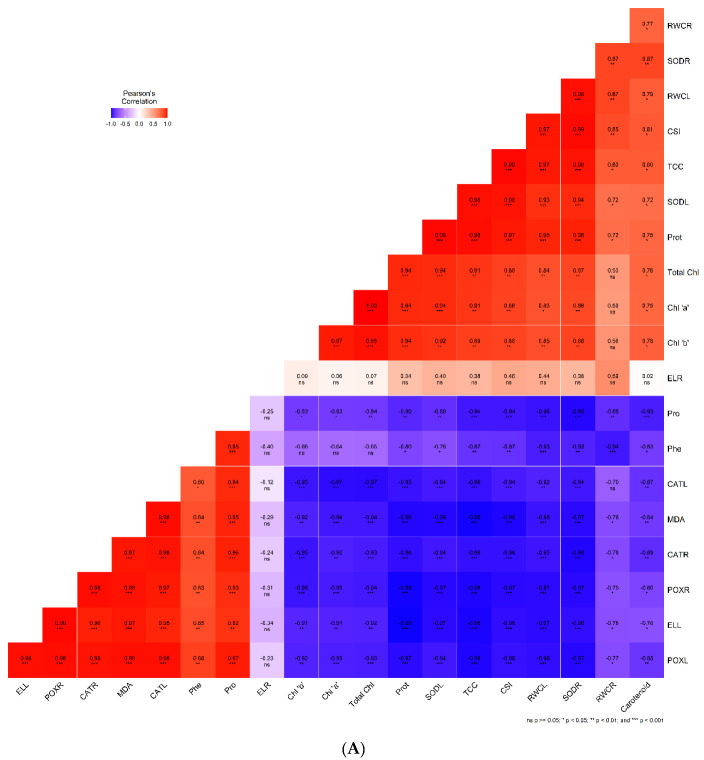

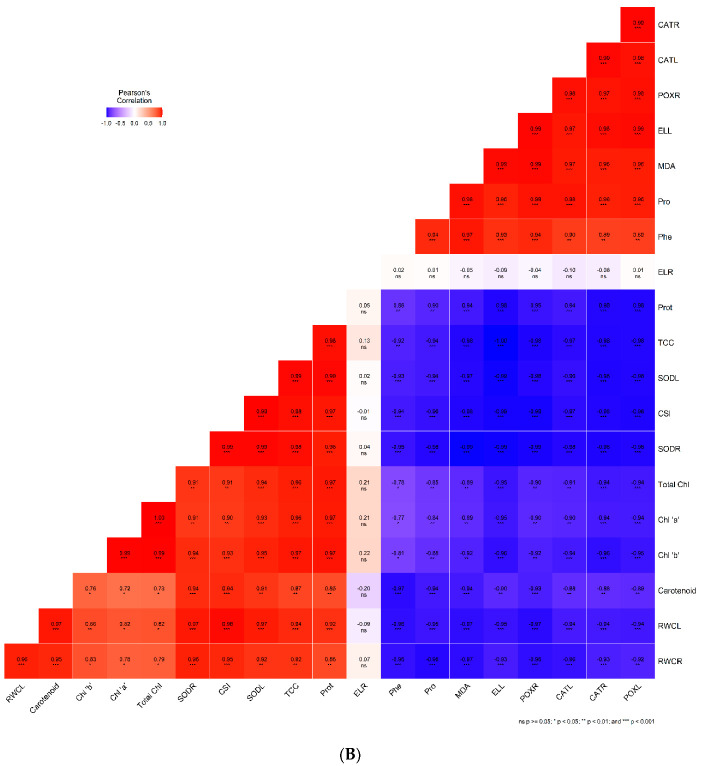
Correlation between different physiological and biochemical parameters, photosynthetic pigments, and antioxidant enzyme activities of KRL-210 (**A**) and WH-1105 (**B**) under different salinity levels with Si treatment (* *p* < 0.05, ** *p* < 0.01, *** *p* < 0.001). The abbreviations used in the figure are as follows: RWCR: relative water content of root, SODR: superoxide dismutase activity of root, RWCL: relative water content of leaves, CSI: chlorophyll stability index, TCC: total carbohydrate content, SODL: superoxide dismutase activity of leaves, Prot: protein content, Total chl: total chlorophyll, chl ‘a’: chlorophyll ‘a’, chl ‘b’: chlorophyll ‘b’, ELR: electrolyte leakage of root, Pro: proline content, Phe: phenol content, CATL: catalase activity of leaves, MDA, CATR: catalase activity of root, POXR: peroxidase activity of root, ELL: electrolyte leakage of leaves, POXL: peroxidase activity of leaves, Carotenoid: carotenoid content.

A Pearson’s correlation graph was created by analyzing the relationship among different parameters, such as physiological, biochemical, and photosynthetic pigments, as well as the antioxidant enzyme activities of KRL-210 and WH-1105 wheat varieties (Figure 5A,B). In both varieties, RWC of leaves and root, SOD activity of leaves and root, CSI, total carbohydrate content, protein content, carotenoid, chlorophyll ‘a’ and ‘b,’ and total chlorophyll were positively correlated to each other at various significant levels (*p* < 0.001, *p* < 0.01, *p* < 0.05) but negatively correlated with proline, phenol, CAT activity of leaves and root, POX activity of leaves and root, MDA content, electrolyte leakage of leaves and vice-versa. Electrolyte leakage of root showed a non-significant correlation with all parameters.

### 2.8. Principal Component Analysis (PCA)

**Figure 6:**
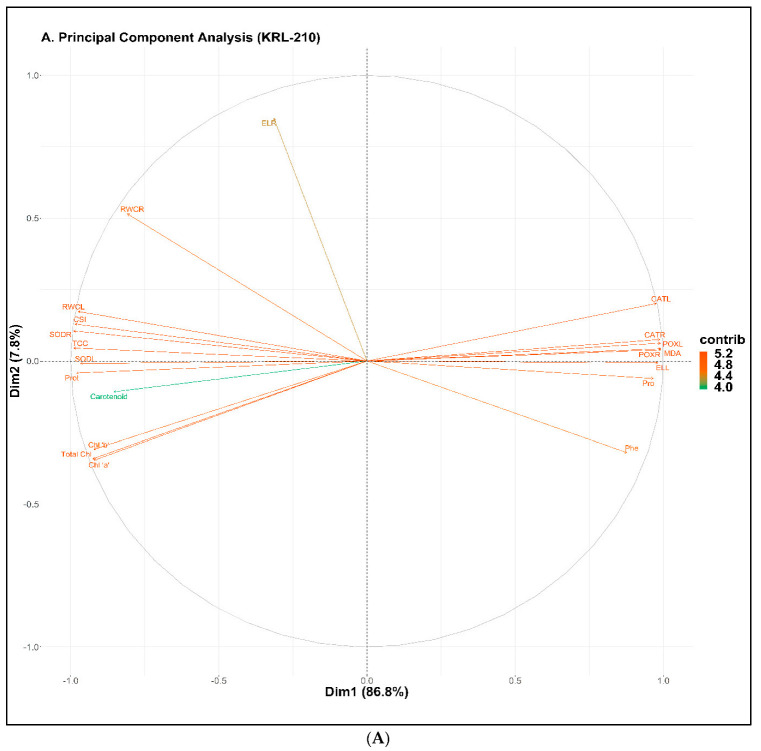

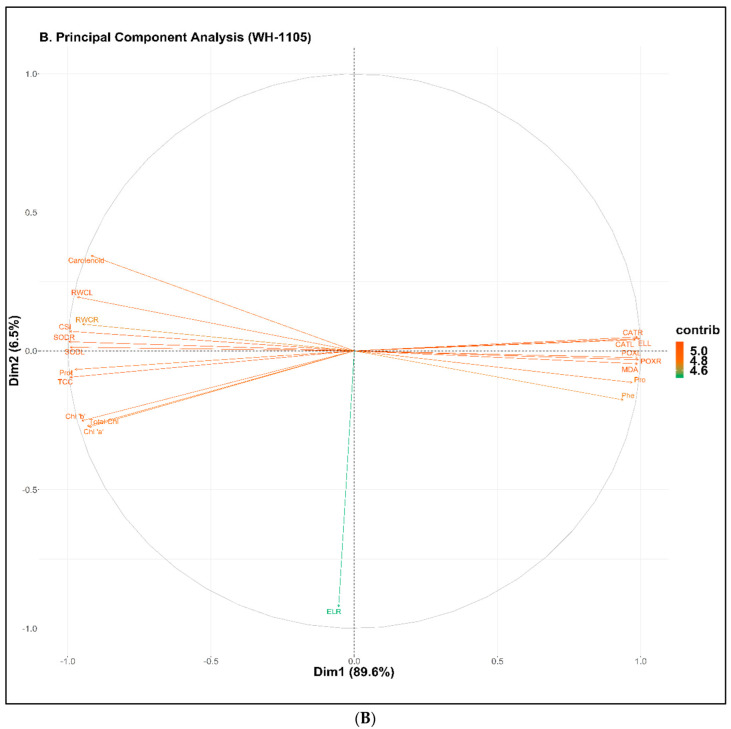
Plots of principal component analysis (PCA) on different studied parameters of KRL-210 (**A**) and WH-1105 (**B**) under different salinity levels with Si treatment. The abbreviations used in the figure are as follows: RWCR: relative water content of root, SODR: superoxide dismutase activity of root, RWCL: relative water content of leaves, CSI: chlorophyll stability index, TCC: total carbohydrate content, SODL: superoxide dismutase activity of leaves, Prot: protein content, Total chl: total chlorophyll, chl ‘a’: chlorophyll ‘a’, chl ‘b’: chlorophyll ‘b’, ELR: electrolyte leakage of root, Pro: proline content, Phe: phenol content, CATL: catalase activity of leaves, MDA, CATR: catalase activity of root, POXR: peroxidase activity of root, ELL: electrolyte leakage of leaves, POXL: peroxidase activity of leaves, Carotenoid: carotenoid content.

A principal component analysis was done to demonstrate the negative impact of various concentrations levels of NaCl stress in two varieties of wheat plants with the exogenous application of Si (Figure 6A,B). For the KRL-210 variety, the first two components, i.e., Dim1 and Dim2, cover around 95% of complete database (Figure 6A), i.e., 86.8% and 7.8%, respectively. In the case of WH-1105, these components comprise about 96% of the overall database (Figure 6B), i.e., 89.6% and 6.5%, respectively. For both varieties, CAT activity in both roots and leaves, MDA content, phenol content, proline content, POX activity in both roots and leaves, and electrolyte leakage in leaves were positively correlated with all other parameters observed in the database except electrolyte leakage in roots. Meanwhile, SOD activity in both roots and leaves, CSI, total protein, and total carbohydrate content, and RWC in leaves were negatively correlated with all the other parameters in both varieties. Electrolyte leakage in roots did not show any correlation with other parameters in either variety. 

## 3. Discussion

Salt is a major abiotic stressor [29]. A high amount of salt in plant root region inhibits water and essential nutrient uptake from soil to plant. This creates water deficit conditions and nutrient imbalance in plants, resulting in osmotic or ionic stress [30]. Many studies have indicated that Si, in the stress condition, enhances plant growth and production by partially alleviating the negative effect of salinity on plants [22].

The RWC of leaves and roots was drastically reduced under salinity (Figure 1A,B). This reduction in the roots and leaves was due to the detrimental effect of salt stress on water absorption from the soil and reduced water availability, which affect the overall water status of the plant [12,29,31]. Likewise, the electrolyte leakage of leaves and roots was also found to be affected by higher salinity levels (Figure 1C,D). The increased concentration of salt stress increases the rate of electrolyte leakage of leaves and roots of wheat varieties. It has been suggested that due to the overproduction of ROS in plant cells, membrane stability or integrity is disrupted, resulting in increased electrolyte leakage under stress conditions [32]. Similar results were obtained in our current investigation. Si mitigated these harmful effects of salinity by increasing the water status and decreasing the rate of electrolyte leakage in plants under salinity by inhibiting the uptake of Na^+^ and enhancing the level of K^+^ [33], which ultimately allowed the plant status, water potential and electrolyte leakage to return to acceptable levels. Similar results have been reported in many plants, showing the role of Si in improving the K^+^/Na^+^ ratio by restricting the influx of Na^+^ [20,34,35,36]. Supplementation of Si was shown to reduce the rate of electrolyte leakage in wheat plants; this may be due to the fact that Si maintains membrane stability and the level of ROS under stressed conditions [34]. The exogenous application of Si alleviates the negative effect of salinity up to a certain level in plants by accumulating in the epidermis of root cells and restricting the inflow of sodium ions, which improve the overall water condition of plants [22]. Moreover, Si supplementation helps prevent water loss and increases cell wall stability due to the deposition of Si in leaves through a decrease in transpiration by forming bonds with cell wall components [37]. The addition of Si under salt stress also increases the hydraulic conductance of roots and upregulates the aquaporin activity in plants, which further improves plant health and increases water levels [38].

In the present research, we have examined chlorophyll a and b, total chlorophyll, carotenoids, as well as CSI under salinity stress and Si treatment (Figure 2A–E). It was found that a high degree of salt stress (120 mM) affected both wheat varieties. The chlorophyll and carotenoid content were drastically reduced with increased salinity when compared with the control. This was probably due to inhibition of ribulose-1,5-bisphosphate enzyme and the structural destruction of the chloroplast and photosynthetic apparatus, ultimately resulting in a decrease in photosynthetic pigments such as chlorophyll, carotenoids, and CSI [20,31]. Similar results were also observed in eggplants under high salt stress, with reductions in photosynthetic pigments, photosynthetic rate, stomatal conductance, and CO_2_ intake [39]. The decrease was found to be more significant in WH-1105 than in KRL-210, showing that the latter is more salt tolerant. Additionally, Si application increased the chlorophyll a, b, and carotenoid contents in wheat plants subjected to salinity. It was also observed that Si application was much more effective under stressed conditions than non-stressed conditions. Si application increases the photosynthetic rate and pigments by suppressing the level of ROS in plant cells, reducing sodium ion toxicity, and maintaining chloroplast structure and function, which is necessary for the photosynthetic process [40,41]. Similarly, in tomatoes, an increase in the concentration of photosynthetic pigments was reported following the application of Si in salt stress [31]. The deposition of Si provides rigidity and erectness to the leaves, allowing them to receive more light for photosynthetic activities, further increasing the formation of chlorophyll pigments [42,43,44].

In our work, proline, phenol, and lipid peroxidation were significantly elevated with increased salt stress levels in both wheat varieties (Figure 3A–C). Supplementation of Si reduced these biochemical changes, which occurred under highly stressed conditions up to a certain limit. Proline and phenol act as osmolytes under salinity stress conditions [29]. The accumulation of these osmolytes protects plants from increased ROS or oxidative stress, an important indicator of stress tolerance [34,35]. Salt stress induces ROS production in plant cells, increasing proline content to scavenge ROS. Si maintains the redox equilibrium by balancing ROS production and proline and phenol levels, resulting in stability in the cellular plasma membrane. Plants produce different types of osmoregulatory compounds under salinity conditions. The application of Si was found to be very helpful in plants to normalize the level of those osmolytes and promote proper functioning of the plant’s processes [45,46]. Likewise, it was found that accumulation of Si in plants reduces the proline and phenol levels by mitigating the adverse effect of salinity [47]. In addition, MDA content was also significantly increased under increased salt stress concentrations. This was probably due to increased oxidative stress in plants and the damaged structure of chloroplasts under salt stress [20]. Si application decreased lipid peroxidation under salinity stress; this may have been due to the role of Si in maintaining the membrane stability or integrity and in regulating osmolyte and ROS levels in the plants [34,48].

The protein and carbohydrate contents were also drastically reduced under salinity (Figure 3D,E). This could have been due to the increased oxidative stress from the overproduction of ROS, resulting in harmful effects in plants like DNA damage, degradation of proteins, and the oxidation of carbohydrates, etc. [49,50,51]. Si enhances protein and carbohydrate concentrations by alleviating the oxidative damage caused by increased ROS during high salinity stress [52]. Similar results were reported by Oraee and Tehranifar [53] in *Bellis perennis*. Si restricts the apoplastic passage of sodium ions from root to shoot by blocking the apoplast. The apoplastic deposition of Si in the form of phytoliths protects plants from over-absorbing sodium ions, resulting in a reduction in oxidative stress and an increase in protein and carbohydrate content [54]. Similarly, in rice and potato plants, it was observed that the accumulation of Si in leaves increases the carbohydrate content under stress conditions [42,55].

CAT, POX, and SOD are the key antioxidant enzymes that neutralize and detoxify plant cells [52]. In our study, the CAT and POX activities were enhanced significantly in leaves and roots under high salinity (Figure 4A–D). According to Abdelaal et al. [20], to deal with the detrimental effects of salt stress and scavenging ROS production in plant cells, plants must defend themselves from oxidative stress. In this regard, they increase antioxidant enzyme activity under salinity. Our findings were supported by reports by Wang et al. [56], Abdelaal et al. [20], and El-Banna and Abdelaal [32]. Si regulates antioxidant enzyme activities and neutralizes oxidative stress in plants by protecting them from cell outbursts [36]. Similarly, applying Si to date palms was shown to reduce the CAT and POX activity under salinity to protect against oxidative damage [57]. SOD activity was found to be reduced at high levels of salt stress in both leaves and roots (Figure 4E,F). This may have been due to a weaker defense mechanism under high salinity; however, Si enhanced the activity of SOD under stress conditions in both varieties. Under stress conditions, antioxidant enzymes regulate the formation of H_2_O_2_ and superoxide (O_2_^*−^). SOD catalyzes the dismutation of H_2_O_2_ and O_2_^*−^. The formation of free radicals is increased in plants under stress conditions [58]. Similar observations were also reported by Mushtaq et al. [52] on wheat varieties under salinity conditions. The KRL-210 variety was less affected by severe salinity stress than WH-1105, indicating its superior salt tolerance.

## 4. Materials and Methods 

### 4.1. Plant Material, Treatments Combinations, and Design

Our experiments were performed on two wheat varieties (WH-1105: salt sensitive and KRL-210: salt tolerant) under stressed and non-stressed conditions with the application of Si treatment at Maharshi Dayanand University, Rohtak. KRL-210 was procured from Central Soil Salinity Research Institute (CSSRI), Karnal, while WH-1105 was obtained from CCS Haryana Agricultural University, Hisar. Seeds were grown in earthen pots containing 7.0 kg of sandy soil, treated with 0.5% dilute sodium hypochlorite solution before sowing to avoid any fungal infection, and later rinsed with deionized water. Four seeds were grown in every pot. Hoagland and Arnon’s [59] nutrient solution was added to the soil at different intervals to provide nutrition after seed germination. The nutrient solution contained CaNO_3_, KH_2_PO_4_, KNO_3_, MgSO_4_, MnCl_2_, H_3_BO_3_, MnSO_4_, ZnSO_4_, CuSO_4_, H_2_MoO_4_, tartaric acid, and ferric citrate. The wheat plants were treated in triplicate with different concentrations of saline water (0, 40, 80, and 120 mM NaCl), from moderate to high salinity, and with Si (Na_2_SiO_3_) alone or in combination with NaCl (0 and 2 mM Si) 25 days after planting. (Table 1) The sampling of leaves and roots was done 50 days after seed germination in order to perform physiological, biochemical, and antioxidant measurements. Pots were placed in a completely randomized design (CRD). There were three plants in each pot after the thinning process, and every treatment had ten replicates of both varieties.

### 4.2. Relative Water Content (Leaves and Root)

The RWC of leaves and roots was measured using the method described by Ghoulam et al. [60]. Fresh leaves and roots from three random plants from each treatment were used as triplicate samples. First, the fresh weight (FW) of all samples was recorded. Samples where then cut into pieces and placed into Petri dishes containing distilled water. Turgid weight (TW) was noted after 4 h of dipping. Samples were then kept in an oven at 85 °C for 2 days to achieve a constant dry weight (DW). The following formula was used to estimate the RWC of leaves and roots (by percentage):

$$\mathrm{RWC}\;\left(\%\right)=\left[\frac{\left(\mathrm{FW}-\mathrm{DW}\right)}{\left(\mathrm{TW}-\mathrm{DW}\right)}\right]\times 100$$

### 4.3. Electrolyte Leakage (Leaves and Root)

The electrolyte leakage (EL) of leaves and roots was measured (by percentage) using the method proposed by Dionisio-Sese and Tobita [61]. Fresh samples were cut into small slices and kept in a test tube with 10 mL of distilled water. After 5 h, the initial electrical conductivity was measured (EC_1_) with a conductivity meter (Microprocessor Conductivity/TDS Meter 1601, ESICO Company). Final electrical conductivity (EC_2_) was noted after heating the samples in a water bath for 1 h. Electrolyte leakage was estimated using the following formula:

$$\mathrm{EL}\;\left(\%\right)=\left(\frac{{\mathrm{EC}}_{1}}{{\mathrm{EC}}_{2}}\right)\times 100$$

### 4.4. Photosynthetic Pigments (Chlorophyll a, b and Carotenoid) 

Fresh leaves were used to estimate the contents of different photosynthetic pigments. The method proposed by Hiscox and Israelstam [62], with slight modifications, was used to estimate chlorophyll content. Briefly, leaves were taken, chopped into fine pieces, and put into DMSO-containing tubes to extract the chlorophyll. After 4–5 h, the absorbance was noted at 665, 645, and 480 nm using dimethyl sulfoxide (DMSO) as a blank with a UV-spectrophotometer (UV 2450, Shimadzu).

### 4.5. Chlorophyll Stability Index (CSI)

The CSI was assessed according to the method proposed by Kaloyereas [63]. Briefly, the CSI was estimated by calculating the difference in percentage of light transmission between heated and non-heated leaf samples. Two sets were prepared. In the first set, 100 mg leaf samples were kept in test tubes containing water at room temperature, while in the second set, leaf samples were kept in a water bath at 55 °C. After 1 h, water was drained from all test tubes, and DMSO was added. Then, the samples were left to sit for 4 h. After complete extraction of chlorophyll pigment, absorbance was measured at 652 nm using a UV-spectrophotometer. The following formula was used to measure CSI:

$$\mathrm{CSI}\;\left(\%\right)=\frac{\mathrm{OD}\;\mathrm{of}\;\mathrm{heated}\;\mathrm{sample}}{\mathrm{OD}\;\mathrm{of}\;\mathrm{non}-\mathrm{heated}\;\mathrm{sample}}$$

### 4.6. Lipid Peroxidation or Malondialdehyde (MDA) Content

The MDA content in leaves was measured using the method proposed by Heath and Packer [64] in order to estimate the level of lipid peroxidation. MDA is the product of lipid peroxidation. The experiment was performed using fresh leaves (0.1 g) which were crushed in 2 mL of 0.1% TCA using a pestle and mortar. The resulting homogenate was centrifuged, and a supernatant was added with 4 mL of 20% TCA containing 0.5% TBA. This mixture was then heated in a water bath at 95 °C. After 30 min, the test tubes were cooled in an ice bath. Absorbance was noted at 532 and 600 nm using a UV-spectrophotometer. The absorbance noted at 532 nm was corrected for unspecific turbidity by deducting the values obtained at 600 nm. The extinction coefficient (155 mM^−1^ cm^−1^) was used to estimate the MDA content, which was expressed as µmol MDA g^−1^ FW.

### 4.7. Total Protein Content

The Lowry method [65] was used to the estimate total protein content. Briefly, 0.1 g of fresh leaf extract was prepared using distilled water as a solvent. The supernatant was collected after homogenization of the mixture and with the addition of 1 mL of deionized water. Then, 5 mL of reagent C, i.e., 2% Na_2_CO_3_ dissolved in 0.1 N NaOH (reagent A), and 0.5% CuSO_4_ in 1% NaKTa (reagent B) in 50:1, was added. Folin-Ciocalteu’s reagent (FCR) was then added to the supernatant. The whole mixture was shaken vigorously with a vortex shaker and kept in the dark for several minutes. Absorbance was noted at 660 nm using a UV-spectrophotometer. The standard curve of absorbance versus protein concentration was used to estimate the protein content in the leaf samples.

### 4.8. Total Proline Content

The proline contents of leaves in wheat were estimated using the methodology proposed by Bates et al. [66]. Fresh leaves (0.1 g) were crushed in 2 mL of aqueous sulphosalicylic acid (3%) with a pestle and mortar. The homogenized mixture was centrifuged, and the supernatant was taken for further experiments. Ninhydrin reagent (1 mL) and glacial acetic acid (1 mL) were added to a test tube containing supernatant. The whole mixture was heated at 100 °C in a water bath. After 10 min, samples were immediately cooled in an ice bath. After cooling, 2 mL toluene was added to all test tubes and shaken with a vortex shaker. The absorbance of proline was noted at 520 nm using a UV-spectrophotometer.

### 4.9. Total Carbohydrate Content

The carbohydrate content was assessed using the method proposed by Yemm and Willis [67]. The standard curve was prepared a graded concentration (20–100 µg ml^−1^) of D-glucose to estimate the carbohydrate content in leaves. Samples of 0.1 g leaves were crushed in 2 mL of 80% ethanol in a pestle and mortar. After centrifugation, 0.2 mL of aliquot was placed into test tubes and the volume was increased to 1 mL with distilled water. Then, 4 mL of anthrone reagent was added and the mixture was boiled in a water bath. After 8–10 min, samples were cooled using an ice bath. Absorbance was recorded at 630 nm using anthrone as a blank.

### 4.10. Total Phenol Content

The phenol content was measured using the Folin-Ciocalteu reagent method [68]. Samples of 0.1 g leaves were crushed in 2 mL of methanol (80%) using a pestle and mortar. The supernatant was collected after centrifugation of the mixture at 10,000 rpm for 15 min. Folin-Ciocalteu reagent and sodium carbonate were then added to the test tube. The absorbance of phenol was recorded at 650 nm using a UV-spectrophotometer.

### 4.11. Antioxidant Enzyme Activity

Fresh leaves and root samples (0.1 g) were taken to measure the activity of antioxidant enzymes like POX, CAT, and SOD. Samples were crushed with 2 mL sodium phosphate buffer (pH 7.8) at 4 °C and centrifuged at 15,000 rpm for 15 min. Antioxidant enzyme activity was analyzed using a supernatant in a spectrophotometer, as explained by Abdelaal et al. [20].

CAT activity was estimated using the Aebi [69] method. The absorbance was noted every 15 s for 1 min at 240 nm.

POX activity was estimated based on the method described by Hammerschmidt et al. [70]. The absorbance was noted every 2 s for 1 min at 470 nm. Enzyme activity was expressed as min^−1^ mg^−1^ FW.

SOD activity was measured using the method proposed by Beauchamp and Fridovich [71]. SOD is of great importance in scavenging O_2_^−^ radicals. The reaction mixture comprised a sodium phosphate buffer containing NBT, Na_2_CO_3_, EDTA, methionine, and riboflavin. Enzyme extract was added to start the reaction. The absorbance of SOD was noted at 560 nm. An enzyme causing 50% inhibition of formazan formation is stated as one unit of SOD activity. It was calculated by the following formula:

Activity (units/mL) = A_C_ − A_T_/A_C_ × 0.5

where A_C_ = absorbance of control and A_T_ = absorbance of treatment.

This activity is reported as Units per mg FW.

### 4.12. Statistical Analysis

The data indicated in the figures are the average values of three replicates and ANOVA analyzed recorded data for two factors. Multiple comparisons were used to determine significant differences between means of treatments using the Tukey’s HSD post hoc test (*p* < 0.05). The graphical representation shown in the figures and PCA plots were created using the RStudio software. The Pearson correlation coefficients between the measured variables of two wheat varieties were also calculated.

## 5. Conclusions

This study deepens our understanding of the beneficial role of Si in alleviating the adverse effects of salinity on two wheat varieties. The WH-1105 variety was more significantly affected by the increasing salt concentration than KRL-210. However, Si application led to increased stress tolerance under high saline conditions. Si increased the RWC, photosynthetic pigments, CSI, protein, and carbohydrate contents, which were significantly reduced under high salt stress conditions in both varieties. Si supplementation enhanced antioxidant enzyme activities by maintaining their levels in plants and reducing the levels of ROS, MDA, and phenol under stressed conditions. Our findings allow us to conclude that Si application is more effective in WH-1105 than KRL-210, showing that Si supports different defensive mechanisms in different plant species. The obtained results will be helpful in increasing the stress tolerance of wheat plants under saline conditions and improving crop production in salty areas through the application of Si as a fertilizer. 

## Figures and Tables

**Table 1:** Experimental layout of different treatments of salinity and Si with different combinations.

	Salt Concentration (mM) 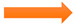	0	40	80	120
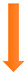 Si Concentration (mM)					
0		0 + 0 (Control)	40 + 0 (S1)	80 + 0 (S2)	120 + 0 (S3)
2		0 + 2 (Si)	40 + 2 (S1 + Si)	80 + 2 (S2 + Si)	120 + 2 (S3 + Si)

NaCl and Si were used individually and in combination for further studies in three replications/treatments.

## Data Availability

All data, figures and results in paper are our own and original.
